# Detecting patterns of atrophy in cognitively impaired individuals using portable, low-field MRI

**DOI:** 10.1162/IMAG.a.1075

**Published:** 2026-01-05

**Authors:** Ava Farnan, Annabel J. Sorby-Adams, Jennifer Guo, John R. Dickson, Liliana Ramirez Gomez, Amanda DeSenna, John E. Kirsch, Joel Smith, Emma Peasley, Jeremy Ford, Seyedmehdi Payabvash, Gordon Sze, Arman Fesharaki-Zadeh, Christopher van Dyck, Matthew S. Rosen, Kevin N. Sheth, J. Eugenio Iglesias, Adam de Havenon, Alberto Serrano Pozo, Teresa Gomez-Isla, W. Taylor Kimberly

**Affiliations:** Department of Neurology, Massachusetts General Hospital and Harvard Medical School, Boston, MA, United States; Center for Genomic Medicine, Massachusetts General Hospital, Boston, MA, United States; Athinoula A. Martinos Center for Biomedical Imaging, Massachusetts General Hospital and Harvard Medical School, Boston, MA, United States; Department of Neurology, Center for Brain & Mind Health, Yale New Haven Hospital and Yale School of Medicine, New Haven, CT, United States; Division of Neuroradiology, Department of Radiology, Massachusetts General Hospital and Harvard Medical School, Boston, MA, United States; Department of Radiology, Colombia University, New York, NY, United States; Division of Neuroradiology, Department of Radiology and Biomedical Imaging, Yale New Haven Hospital and Yale University School of Medicine, New Haven, CT, United States

**Keywords:** low-field MRI, dementia, artificial intelligence, segmentation

## Abstract

Low-field MRI (LF-MRI) is an emerging neuroimaging approach for evaluating patients with dementia, offering greater accessibility and lower cost, albeit with reduced image resolution. In this study, we deployed LF-MRI in an outpatient clinic and analyzed images with a multi-functional artificial intelligence (AI) algorithm (WMH-SynthSeg) to generate segmentation volumes of 16 brain regions. We validated the accuracy of the quantifications compared with conventional, high-field (HF) MRI in healthy volunteers and subsequently applied the algorithm to aged subjects with mild cognitive impairment (MCI) or dementia due to Alzheimer’s disease (AD), and to similarly aged subjects with cognitive impairment and vascular comorbidities (VC). Agreement between HF- and LF-derived brain volumes was high across cohorts and brain regions, with the highest correlations in the cortex, white matter, lateral ventricles, third ventricle, caudate, and amygdala (all r > 0.80, p < 0.001). The MCI and AD cohorts showed regional atrophy relative to the VC cohort, including the cortex, hippocampus, amygdala, putamen, and nucleus accumbens (all p < 0.001) but not the caudate, ventral diencephalon, and fourth ventricle (p > 0.05). Taken together, LF-MRI paired with an AI segmentation algorithm can generate brain volumes comparable with those derived from conventional MRI, allowing for differentiation between VC and AD/MCI subgroups. Our findings demonstrate that LF-MRI could be used at the point-of-care for evaluation of patients with dementia of different etiologies.

## 1 Introduction

The number of people living with dementia globally is expected to increase from 57 million cases in 2019 to 157 million cases by 2050 (Nichols et al., 2022). Alzheimer’s Disease (AD), the leading cause of dementia, is characterized by the accumulation of amyloid β (Aβ) plaques and hyperphosphorylated tau in the brain, which leads to synaptic dysfunction and neuronal loss. Patients experience progressive cognitive decline (Ma et al., 2016), where widespread neuronal atrophy (Fox et al., 1999), particularly in regions such as the hippocampus and neocortex, are important biomarkers of the disease (Sorby‐Adams, Guo, Laso, et al., 2024). Conversely, vascular cognitive impairment (VCI), the second most prevalent form of dementia, arises due to underlying vascular comorbidities resulting in impaired cerebral blood flow and tissue injury in the deep and periventricular white matter (Alber et al., 2019; Logue et al., 2011; Sanders et al., 2025). There is frequently a complex interplay observed between VCI and AD where vascular dysfunction can exacerbate Aβ and tau pathology, while AD can compromise vascular integrity, together accelerating cognitive decline (Rundek et al., 2022). This dynamic contributes to the manifestation of mixed dementia, where both vascular and neurodegenerative factors interact to drive disease progression (Chui et al., 2006; Custodio et al., 2017; Zekry et al., 2002).

Magnetic resonance imaging (MRI) plays an integral role in the evaluation of patients with dementia of heterogeneous etiology. It first facilitates the exclusion of alternative causes of cognitive impairment and subsequently enables the detection of regional brain atrophy and vascular contributions to cognitive decline, which frequently manifest as white matter hyperintensities (WMH) in the periventricular and deep white matter (Sorby‐Adams, Guo, Laso, et al., 2024). Conventional, high-field MRI (HF-MRI) is the gold standard for clinical assessment due to its superior resolution and signal-to-noise ratio (SNR). However, HF-MRI requires fixed installations, substantial power requirements, and a reliable source of cryogens, the combination of which limits accessibility and increases cost. Recent advancements in portable, low-field MRI (LF-MRI) provide an alternative approach that has the potential to increase access due to its lower cost, simpler operation, and increased safety factor (Kimberly et al., 2023; Zhao et al., 2024). However, LF-MR images have lower resolution and reduced SNR which pose a challenge for downstream tasks such as segmentation and volumetric quantification of brain regions and WMH (Rundek et al., 2022). Integration of artificial intelligence (AI) with LF-MRI can overcome this limitation by enhancing resolution and enabling accurate segmentation for quantitative analysis of brain structures (Iglesias et al., 2023; Sorby‐Adams, Guo, Laso, et al., 2024).

In this study, we sought to build upon our prior work (Iglesias et al., 2023; Sorby‐Adams, Guo, Laso, et al., 2024) by evaluating a multi-task AI algorithm that can jointly segment brain regions and WMH solely relying on T2 fluid-attenuated inversion recovery (FLAIR) images. We first aimed to confirm the accuracy of the algorithm by comparing paired LF- with HF-MRI-derived volumes. We then evaluated differences in WMH and brain regions in patients with cognitive impairment due to AD (including those with mild cognitive impairment [MCI] and mild-to-moderate dementia) when compared with those with cognitive impairment due to vascular factors.

## 2 Methods

### 2.1 Study design

We retrospectively studied participants who were enrolled at the Massachusetts General Hospital (MGH) and Yale New Haven Hospital as part of an observational study. Three cohorts were included: healthy volunteers (HV), subjects with vascular comorbidities (VC), and patients with MCI or mild-to-moderate dementia due to AD. For healthy volunteers, subjects with no cardiovascular risk factors or history of neurological disease were enrolled at MGH between December 2022 and July 2023. Patients with a clinical diagnosis of MCI or dementia due to AD and varying levels of cardiovascular risk factors were enrolled from the MGH Outpatient Neurology Clinic between February 2023 and August 2024. Individuals with at least one vascular risk factor were enrolled at Yale New Haven Hospital (YNHH) between December 2021 and July 2022. These participants were observed to have cognitive impairment based on cognitive assessment scores conducted after enrollment.

Demographic and medical history, including cognitive performance on the Montreal Cognitive Assessment (MoCA) and the presence of cardiovascular risk factors, were collected for all participants in the VC and MCI/AD cohorts. Cardiovascular risk factors included a history of hypertension or treatment with antihypertensive medications, a history of hyperlipidemia or statin therapy, a history of type 2 diabetes mellitus or antidiabetic therapy, a history of congestive heart failure, current smoking, and atrial fibrillation (de Havenon et al., 2023). AD pathology was confirmed in all AD/MCI participants through positive cerebrospinal fluid and/or positron emission tomography biomarkers, confirming brain Aβ accumulation based on clinical standard of care thresholds for the respective tests (Jack et al., 2024). Apolipoprotein ε4 (APOE4) status was collected for MCI/AD participants with available genotyping. The VC participants were enrolled in the YNHH emergency room based on vascular comorbidities rather than memory or cognitive complaints, therefore, determination of amyloid status was not performed as a part of their clinical evaluation. Exclusion criteria included an inability to lay flat, weight >400 lbs, pregnancy, active electronic implants, and age <18 years. Informed consent was obtained for all participants under Mass General Brigham and/or Yale University Institutional Review Board approval.

### 2.2 Image acquisition

All participants underwent T2 FLAIR acquisition on a 0.064 T, LF-MRI device (Hyperfine Inc.). HV and AD/MCI participants were scanned on v1.9 hardware and VC participants were scanned on v1.6 hardware (de Havenon et al., 2023). Sequence parameters across hardware and software versions are reported for each cohort in Supplementary Table S1. HF-MRI T2 FLAIR images acquired for clinical care or research purposes were used for comparison, which was considered the gold standard (AD [n = 10 at 1.5 T; n = 12 at 3 T], MCI [n = 15 at 1.5 T; n = 13 at 3 T], VC [n = 11 at 1.5 T; n = 11 at 3 T], and HV [n = 25 at 3 T]). All images acquired were processed centrally through FreeSurfer (Fischl et al., 2002) (v7.3.2) using AI algorithm WMH-SynthSeg.

### 2.3 Machine learning algorithm

WMH-SynthSeg (Sorby‐Adams, Guo, De Havenon, et al., 2024) is an AI algorithm optimized for the simultaneous segmentation of anatomical regions of interest and white matter T2 hyperintense regions consistent with WMH. The details of the algorithm have been published previously (Sorby‐Adams, Guo, Laso, et al., 2024). Briefly, the algorithm builds on the architecture of the previously reported pipelines LF-SynthSR and SynthSeg (Laso et al., 2023; Rundek et al., 2022; Sorby‐Adams, Guo, Laso, et al., 2024) but introduces two important advancements. First, the training data are sourced from public datasets (Human Connectome Project, Alzheimer’s Disease Neuroimaging Initiative, WMH segmentation challenge) and includes scans with atrophy, WMH lesions, and corresponding manual segmentation masks. Second, WMH-SynthSeg uses a multi-task U-net to generate predictions simultaneously so that the algorithm generates both regional and WMH segmentation volumes. The algorithm accepts inputs of varying resolution, enabling analysis of both HF-MRI at 1.5 or 3 T and LF-MRI scans at 0.064 T.

### 2.4 Image analysis

For all cohorts, LF and HF T2 FLAIR images were manually inspected for quality before conversion to NIfTI open file format and segmentation with WMH-SynthSeg into 33 brain regions. For all bilateral structures, the left and right volumes were averaged, except for the left and right lateral ventricle which were combined. The total white matter (WM) volume was calculated by summing normal-appearing WM and WMH. This yielded a total of 16 separate volumes.

To evaluate the accuracy of the LF-MRI-derived segmentations, volumes from each subject were compared with HF-MRI counterparts with quantitative metrics including Pearson’s correlation coefficients, the absolute symmetrized percent difference (ASPD), and Dice similarity coefficient, as previously described (Sorby‐Adams, Guo, Laso, et al., 2024). The Dice coefficient was calculated by first co-registering HF scans to LF-MR images using a nonlinear approach in NiftyReg (Siebert et al., 2022), with a grid spacing of 30 mm and a computed kernel of 5 mm, and subsequent WMH-SynthSeg segmentation of the co-registered image. Previous work (Sorby‐Adams, Guo, De Havenon, et al., 2024) identified these parameters as the optimal approach to co-registration. Prior to comparisons between cohorts, segmentation volumes were corrected for total intracranial volume to account for differences in head size.

### 2.5 Statistical analyses

Statistical analyses were performed using STATA v18 (StataCorp, College Station, Texas, USA) and RStudio v4.4.2 (R Core Team 2024). Pearson rho (r) correlations were performed with Fisher’s transformation to derive 95% confidence intervals (CI). Comparisons between cohorts were evaluated using the Kruskal–Wallis test with Dunn’s post hoc test. Pearson’s correlations were defined as strong (r > 0.7), moderate (r ≤ 0.7 to < 0.5), fair (r ≤ 0.5 to ≤ 0.3), or poor (r < 0.3). A Dice of 1 and ASPD of 0 indicated perfect agreement in spatial overlap and volume between LF and HF counterparts, respectively. Bland–Altman plots were also generated to evaluate agreement between LF and HF measurements, with mean differences and 95% limits of agreement displayed. To account for potential confounding variables, ordinal logistic regression analyses were performed with mean-centered and unit-standardized values. The groups were ordered according to disease severity so that VC<MCI<AD. Receiver operating characteristic (ROC) analyses were performed to estimate area under the curve (AUC) values with 95% CI, followed by pairwise comparisons of AUCs across field strengths. To account for multiple comparisons across the 16 brain regions, a Bonferroni correction was applied which resulted in a significance threshold of p < 0.0031. Data are reported as 95% CI, interquartile range [IQR], relative risk ratio (RRR), and odds ratios (OR) where applicable. A p < 0.05 was considered statistically significant.

## 3 Results

### 3.1 Cohort characteristics

We studied healthy volunteers (n = 25), patients with vascular comorbidities and cognitive impairment (n = 22), and patients with MCI (n = 28) or AD (n = 22). HF-MRI scans were acquired within 2 months [IQR 0, 6] of the LF-MRI for all subjects. Due to incomplete coverage of the posterior fossa, cerebellar segmentation volumes were excluded from the HV cohort (n = 2), VC cohort (n = 6), and the MCI/AD cohort (n = 14). All other brain structures had complete supra- and infra-tentorial acquisition and segmentation volumes derived. The demographic information for all participants included in analyses is reported in Table 1.

**Table 1.** Cohort characteristics.

	Healthy volunteer (n = 25)	Vascular comorbidities (n = 22)	Mild cognitive impairment (n = 28)	Alzheimer’s disease (n = 22)
Age, years, mean ± SD	29 ± 7	64 ± 8	72 ± 7	71 ± 9
Sex, n (%)				
Female	15 (60%)	11 (50%)	14 (50%)	5 (23%)
Male	10 (40%)	11 (50%)	14 (50%)	17 (77%)
Race, n (%)				
White	18 (72%)	12 (55%)	27 (96%)	22 (100%)
Asian	5 (20%)	1 (5%)	0 (0%)	0 (0%)
Black or African American	1 (4%)	7 (32%)	0 (0%)	0 (0%)
Other	1 (8%)	2 (9%)	1 (4%)	0 (0%)
Ethnicity, n (%)				
Hispanic or Latinx	0 (0%)	4 (18%)	1 (3%)	1 (5%)
Not Hispanic or Latinx	21 (84%)	18 (82%)	24 (86%)	21 (95%)
Other	4 (16%)	0 (0%)	3 (11%)	0 (0%)
MoCA, mean ± SD	-	22 ± 4	21 ± 5	18 ± 6
APOE ε4 status, n (%)				
Non-carrier	-	-	8 (29%)	10 (45%)
Heterozygous	-	-	13 (46%)	5 (23%)
Homozygous	-	-	2 (7%)	4 (18%)
Unknown	-	-	5 (18%)	3 (14%)
Vascular risk factors, n (%)				
Hypertension	-	18 (82%)	14 (50%)	10 (45%)
Hyperlipidemia	-	17 (77%)	18 (64%)	12 (55%)
Diabetes mellitus	-	10 (45%)	0 (0%)	3 (14%)
Atrial fibrillation	-	4 (18%)	0 (0%)	1 (5%)
Congestive heart Failure	-	2 (9%)	1 (4%)	0 (0%)
Current smoking	-	3 (14%)	0 (0%)	0 (0%)
2 or more risk factors	-	19 (86%)	14 (50%)	6 (41%)

APOE—apolipoprotein E, MoCA—Montreal cognitive assessment, SD—standard deviation.

### 3.2 Accuracy of LF-MRI segmentation

**Fig. 1.**
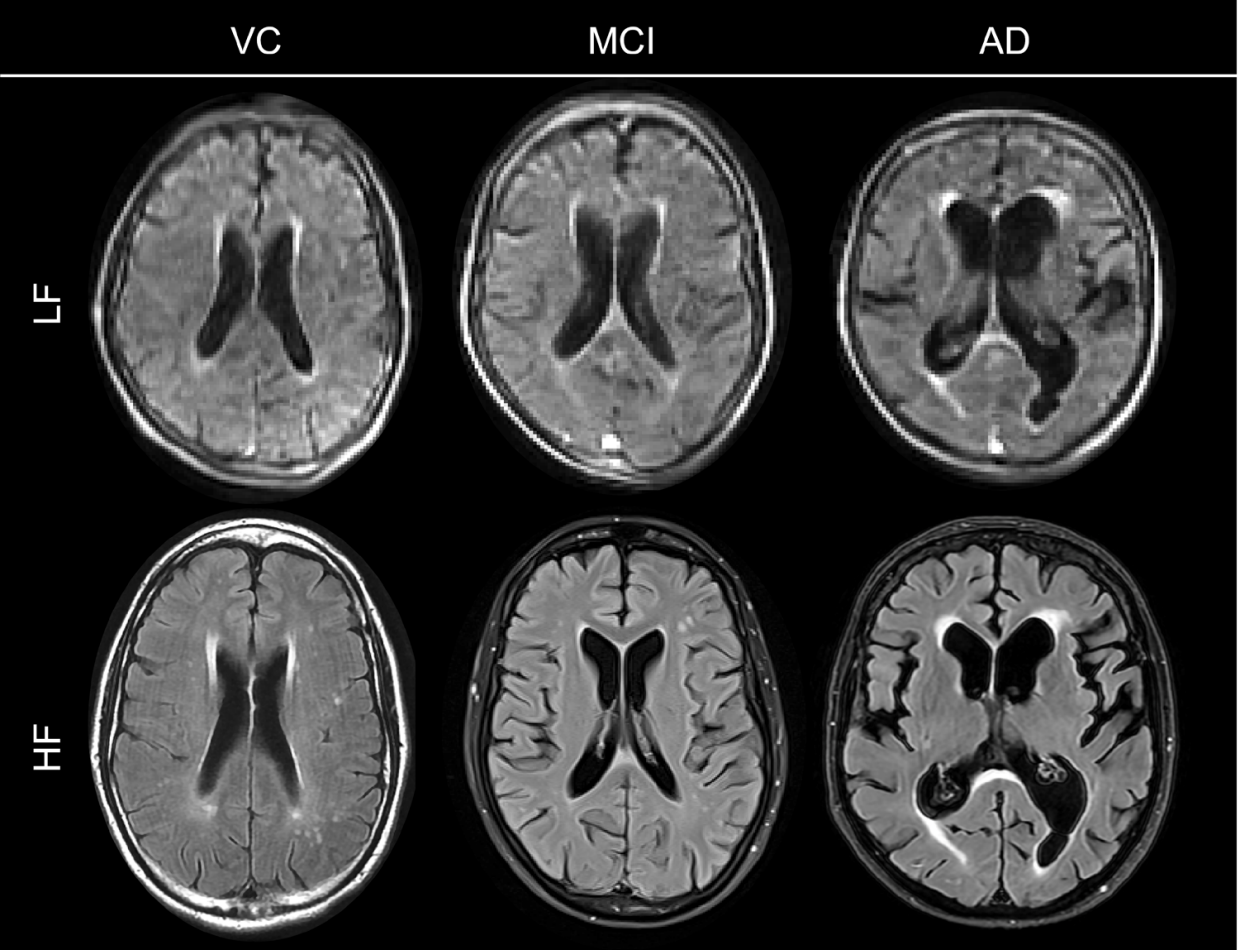
Representative low- and high-field T2 FLAIR images across diagnostic groups. Axial low-field (LF; top row) and high-field (HF; bottom row) FLAIR images from three participants representing typical imaging features of each cohort: vascular comorbidities (VC, left), mild cognitive impairment (MCI, middle), and Alzheimer’s disease (AD, right). LF images demonstrate visible WMH and ventricular enlargement, though with reduced resolution compared with HF.

Previously, we had shown that accurate brain segmentation volumes could be generated from co-registered LF-MRI T1- and T2-weighted images, or from each sequence separately (Sorby‐Adams, Guo, De Havenon, et al., 2024; Sorby‐Adams, Guo, Laso, et al., 2024). However, since T2 FLAIR sequences are commonly acquired in clinical settings and are the optimal sequence for WMH measurement, we focused on T2 FLAIR images from LF-MRI for this study. To generate regional and WMH volumes, we implemented WMH-SynthSeg to jointly derive the volumes of interest based on T2 FLAIR image inputs. Example images at high- and low- field are shown in Figure 1.

**Fig. 2.**
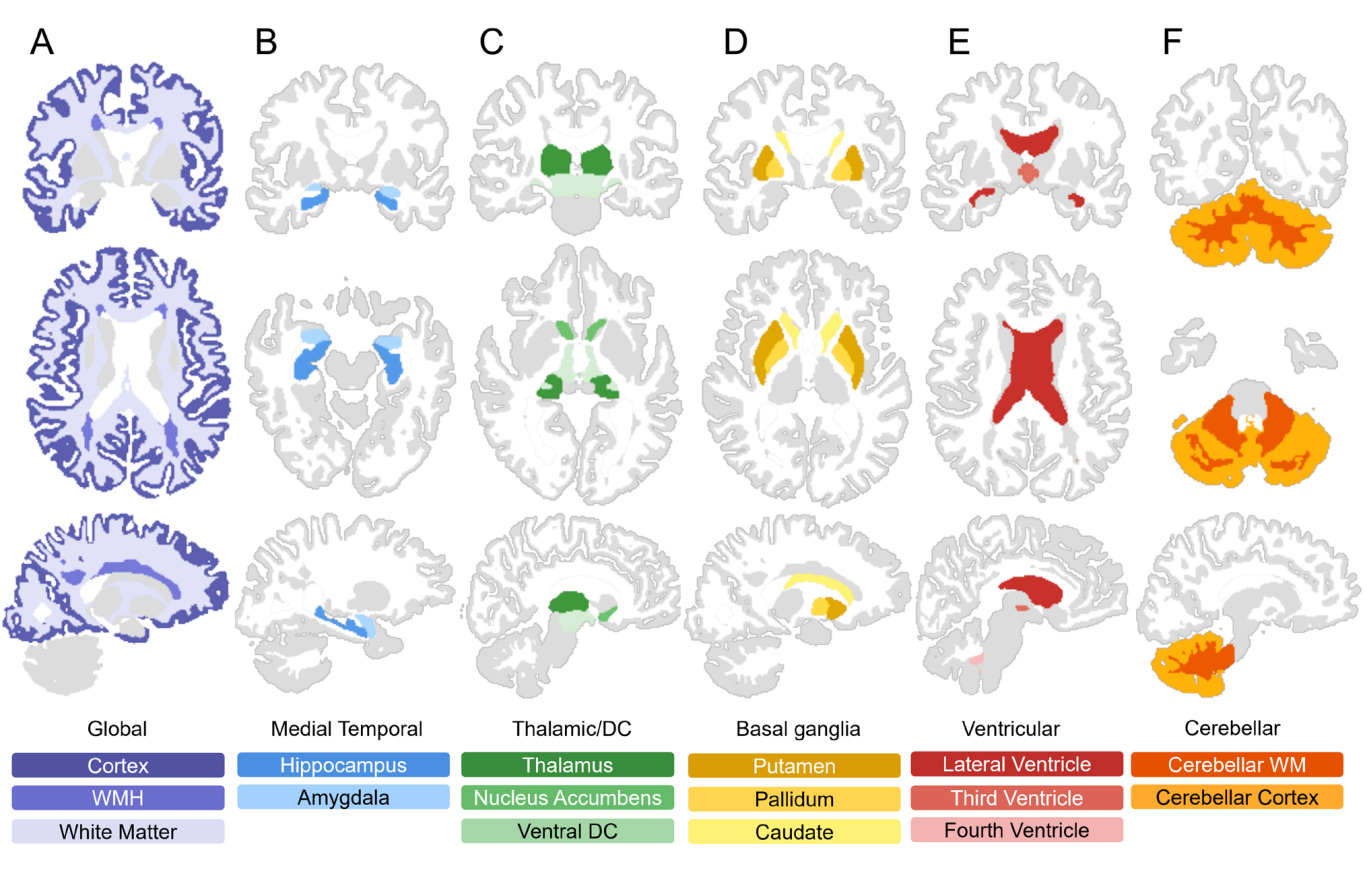
Visualization of brain structures segmented by WMH-SynthSeg. Representative coronal, sagittal, and axial views of brain regions segmented by the WMH-SynthSeg algorithm. Each panel (A–F) highlights structures by group.

**Fig. 3.**
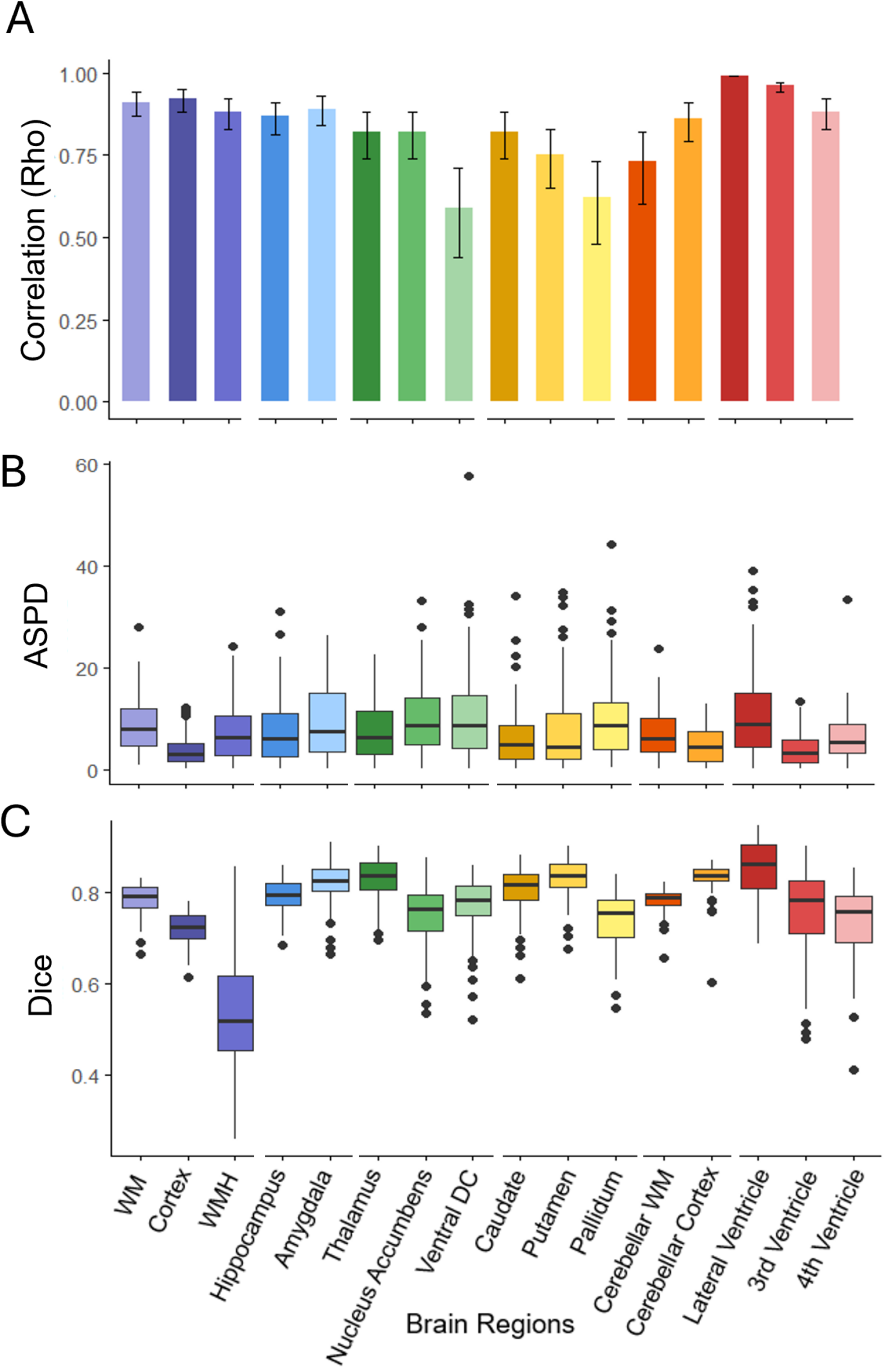
Validation of WMH-SynthSeg segmentations between low- and high-field MRI. Agreement of WMH-SynthSeg brain region segmentations between low-field (LF) and high-field (HF) MRI using (A) Pearson correlation (r) coefficients (mean ± 95% CI), (B) Absolute Symmetrized Percent Difference (ASPD), and (C) Dice similarity coefficients. Each brain region is grouped by tissue class and color coded to match the labeling scheme in Figures 1 and 2.

Using WMH-SynthSeg, we generated 16 brain volumes of interest summarized in Figure 2, with the left and right segmented volumes reported individually in Supplementary Tables S2 and S3. To evaluate the accuracy of segmentation volumes generated, we first examined the correlation between HF- and LF-FLAIR counterparts (Fig. 3A and Supplementary Table 2). All regions had correlations of r > 0.59 (p < 0.001) including the normal-appearing WM (r = 0.91; 95% CI 0.87, 0.94), cortex (r = 0.92; 95% CI 0.88, 0.95), and WMH (r = 0.88; 95% CI 0.83, 0.92). The subcortical structures with the highest correlation included the hippocampus (r = 0.87; 95% CI 0.81, 0.91), amygdala (r = 0.89; 95% CI 0.84, 0.93), and thalamus (r = 0.82; 95% CI 0.74, 0.87), whereas the ventral diencephalon showed the lowest (r = 0.59; 95% CI 0.44, 0.71). The time interval between the HF and LF scans was examined as a potential confounder and found to have no effect on the significance or strength of the observed correlation analyses (all p < 0.001). The full set of correlations are provided in Supplementary Tables S4–S7 for each cohort and the Bland–Altman plots for all cohorts are reported in Supplementary Figure S1.

Next, we examined the agreement in volume for each structure based on the percentage difference relative to gold standard (i.e., ASPD; Fig. 3B). The structures with the lowest error included the WM with an ASPD of 1.31% [IQR 0.67, 2.08] and the cortex with an ASPD of 3.15% [IQR 1.44, 6.45]. The structure with the highest percentage difference between field strengths was the putamen with an ASPD of 10.07% [IQR 3.69, 18.25].

To evaluate the degree of spatial overlap between the segmentation masks derived from LF to HF scans, we examined the Dice similarity coefficient for each structure. The Dice coefficients were > 0.72 for all structures except for WMH, which was 0.52 [IQR 0.45, 0.62], as shown in Figure 3C and Supplementary Table S4. All ASPD and Dice scores are reported for each cohort in Supplementary Tables S5–S7.

### 3.3 Atrophy differences between the VC and MCI/AD cohorts

**Fig. 4.**
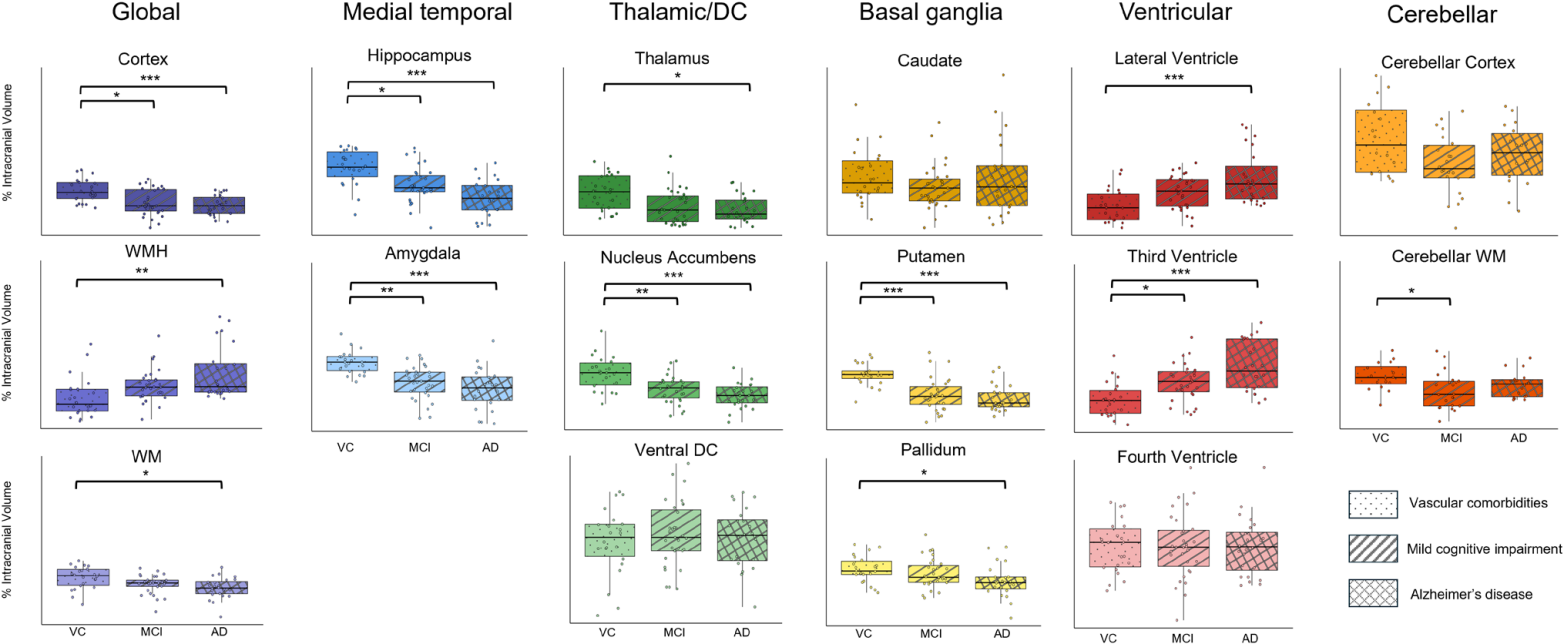
Comparison of low-field (LF) segmented brain region volumes across cohorts. Box plots comparing segmented brain volumes between the vascular comorbidities (VC), mild cognitive impairment (MCI), and Alzheimer’s disease (AD) cohorts. Each brain region is grouped by tissue class and color coded to match the labeling scheme in Figures 1 and 2. Individual data points are overlaid on each box plot to show the distribution of individual measurements. All volumes are adjusted by intracranial volume and reported as percentage of intracranial volume. Significance is denoted as *p < 0.05, **p < 0.01, and ***p < 0.001.

Having validated the accuracy of the LF-MRI-derived values, we next assessed for differing patterns of atrophy between the VC, MCI, and AD cohorts (Fig. 4). The cortical gray matter, hippocampus, amygdala, and nucleus accumbens were smaller in MCI and AD subjects than in VC subjects (p < 0.05). Consistent with prior HF-MRI studies, additional subcortical structures showed groupwise differences. The thalamus and pallidum demonstrated atrophy in AD subjects only (p < 0.05), while the putamen showed atrophy in both MCI and AD subjects (p < 0.001). Concordant with atrophy, the lateral and third ventricles but not the fourth ventricle were larger in the MCI and AD subjects (p < 0.001). Additional structures that showed modest or no decrease in AD/MCI relative to the VC cohort included the caudate, ventral diencephalon, and cerebellum (p > 0.05).

As a marker of vascular pathology, we found that WMH volume was increased (p < 0.01) while WM volume was decreased in the AD cohort relative to VC cohort (p < 0.01). There was no difference in WM or WMH volume observed between the VC and MCI cohorts (p > 0.05).

Similar findings were observed for the HF data, and these results are provided in the Supplementary Information (Supplementary Fig. S2). To assess the predictive value of the segmented volumes, we generated ROC curves for the classification of VC versus MCI/AD cohorts which are reported in the Supplementary Information (Supplementary Fig. S3). The AUC values derived from these curves were similar at low field and high field for a majority of the volumes and are reported in Supplementary Table S8.

### 3.4 Multivariable analysis

Next, we evaluated which structures were independently associated with each cohort by adjusting for age and sex as potential confounders in an ordinal regression analysis where VC<MCI<AD. Smaller volumes of the nucleus accumbens, amygdala, hippocampus, and putamen (OR < 1, p < 0.0002) were associated with a higher likelihood of being in the MCI and AD cohort. Conversely, greater volumes of lateral ventricle, third ventricle, and WMH were associated with an increased likelihood of being in the MCI and AD cohort (OR > 1, p ≤ 0.003, see Table 2 for all results). Given our sample size, we performed logistic regression with backward selection as an exploratory analysis which identified two potential structures that were independently associated with cohort groupings. Specifically, larger lateral ventricle volumes were associated with greater odds of belonging to the AD cohort (RRR = 2.57, p = 0.02), while larger putamen volumes were associated with the VC cohort (RRR = 4.93, p = 0.002).

**Table 2.** Multinomial ordinal regression analysis between cohorts.

	Odds ratio	p value
Global		
WM	0.52	0.024
Cortex	0.47	0.004
WMH	2.40	0.003
Medial temporal		
Hippocampus	0.25	<0.001
Amygdala	0.29	<0.001
Thalamic		
Thalamus	0.48	0.012
Nucleus accumbens	0.26	<0.001
Ventral DC	1.29	0.262
Basal ganglia		
Caudate	1.05	0.828
Putamen	0.28	<0.001
Pallidum	0.66	0.098
Cerebellum		
Cerebellar WM	0.85	0.576
Cerebellar cortex	0.80	0.446
Ventricles		
Lateral ventricle	2.93	<0.001
Third ventricle	3.19	<0.001
Fourth ventricle	0.79	0.277

DC—diencephalon, WM—white matter, WMH—white matter hyperintensities, VC is the reference cohort.

## 4 Discussion

This study assessed the accuracy and clinical utility of LF-MRI combined with an AI algorithm to assess structural biomarkers associated with cognitive impairment of varying etiologies in an outpatient clinic setting. We demonstrate high agreement between volumes derived from LF- and HF-MRI counterparts. Three measures of agreement were used for validation, including Pearson’s correlation, ASPD, and Dice scores. Comparable volumes were observed across field strengths, supporting previously reported validation analysis (Sorby‐Adams, Guo, Laso, et al., 2024). However, Dice scores were lower for WMH volumes than for other brain regions, likely because the small volume of WMH increases sensitivity to spatial mismatches. We further showed that patients with dementia due to AD demonstrated atrophy in the hippocampus, amygdala, and cortex, and larger lateral ventricle volumes relative to those with cognitive impairment due to vascular risk factors.

Portable, LF-MRI technologies offer a cost-effective, accessible (Kimberly et al., 2023) alternative to HF-MRI, enabling scanning in outpatient settings. As brain atrophy is a recognized biomarker of AD, while WMH is concordant with underlying vascular disease, and both are biomarkers of dementia of mixed etiology, we investigated the utility of LF-MRI in differentiating between dementia subtypes using brain volumes derived from an AI algorithm. Cortical atrophy was observed globally in patients with MCI and dementia due to AD when compared with VC patients, accompanied by regional atrophy in the medial temporal lobe, including a significant reduction in volume of the hippocampus and amygdala, consistent with known biomarkers of AD (Barnes et al., 2006; Cavedo et al., 2011; Coupé et al., 2019; Cuénod et al., 1993; De Jong et al., 2008; De Leeuw et al., 2004; Deweer et al., 1995; Frisoni et al., 2006; Henneman et al., 2009; Krasuski et al., 1998; Laakso et al., 1995; Pini et al., 2016; Ridha et al., 2008; Shi et al., 2009; Van der Flier, 2005; Whitwell et al., 2005). In keeping with diffuse neuronal loss, we also saw significant ventricular enlargement in the lateral and third ventricles in those with AD/MCI, while volume of the fourth ventricle remained constant. Patients with AD also demonstrated increased WMH volumes, and decreased WM volume, relative to VC patients. Greater WMH volume in the AD/MCI cohort was unexpected given the VC cohort has a greater burden of vascular risk factors. Prior studies have, however, shown that WMH burden increases in those with MCI and AD compared with those without positive Aβ markers (De Leeuw et al., 2004; Garnier-Crussard et al., 2022, 2023; Van Den Berg et al., 2018), suggesting that Alzheimer’s pathology itself may contribute to white matter changes. Additionally, mixed dementia is commonly observed in individuals with dementia due to AD (Alber et al., 2019; Rundek et al., 2022; Zekry et al., 2002) and is likely present in our AD/MCI cohort, potentially contributing to these findings. However, there are several postulated causes for WMH across different populations, which makes direct cohort comparisons challenging and remains an avenue for future investigation (De Leeuw et al., 2004; Garnier-Crussard et al., 2022, 2023; Van Den Berg et al., 2018).

Beyond the established biomarkers of AD and VCI, we extended our analysis to include regions such as the basal ganglia (Cho et al., 2014; Jernigan et al., 2001; Liu et al., 2010; Madsen et al., 2010; Pievani et al., 2009; Roh et al., 2011; Tang et al., 2014; Yi et al., 2016) and thalamus (Callen et al., 2001; Forno et al., 2023; Tang et al., 2014). In our analysis, we demonstrate atrophy in the thalamus and nucleus accumbens in those with AD relative to the VC cohort, while the ventral diencephalon showed no change in volume between cohorts. The caudate showed no atrophy between disease states, in concordance with prior studies suggesting minimal involvement in AD pathology (Roh et al., 2011; Yi et al., 2016), though other research highlights its role in advanced disease stages (Jernigan et al., 2001; Madsen et al., 2010; Tang et al., 2014). Volume of the putamen was significantly reduced in both MCI and AD when compared with the VC cohort, and volume of the pallidum was reduced in those with AD. While prior studies report atrophy in the putamen (Coupé et al., 2019; Roh et al., 2011; Tang et al., 2014), a reduction in volume of the pallidum is an uncommon observation in patients with AD (Cho et al., 2014; Roh et al., 2011). Thus, the pallidum atrophy reported herein could be due to the slightly older average age of the AD relative to VC cohort. Furthermore, in exploratory analysis, our study identified the putamen and lateral ventricles as potential structures to show the differential atrophy pattern between VC and AD, while structures such as the pallidum were not significant.

Further validation in larger and more diverse cohorts, including longitudinal analyses, is necessary to establish LF-MRI and AI algorithms such as WMH-SynthSeg for tracking neurodegenerative disease progression. Subtle differences in low-field-derived images can be influenced by various factors that include scanner hardware, scanner software, the local electromagnetic environment, temperature fluctuations, and the patient’s position within the fixed head coil. While these are important considerations, differences due to these factors are smaller in magnitude than the biological differences reported in our comparison cohorts. The accuracy of LF images relative to HF counterparts is also influenced by the size of the brain volumes being investigated, with larger structures demonstrating more reliable results, highlighting that some clinical scenarios may be better suited to LF-MRI use than others, and that its scalability remains an area for further investigation. In addition, future studies could include an age-matched cognitively normal cohort for comparison with those with cognitive impairment. Notably, the HV and MCI/AD cohorts were recruited at one site, while the VC cohort was recruited at another, introducing a potential site-related confounder. Future multi-site studies that focus on contemporaneous recruitment of cohorts across participating sites will be important for broader validation.

### 4.1 Conclusions

This study evaluated LF-MRI and the WMH-SynthSeg AI algorithm to segment and subsequently quantify brain volumes. These measurements were validated across multiple cohorts including patients with cognitive impairment due to vascular comorbidities and Alzheimer’s pathology. This study underscores the potential of combining AI with LF-MRI to make neuroimaging more accessible, particularly for those with cognitive impairment. Such innovations pave the way for integrating accessible imaging solutions into routine clinical care.

## Supplementary Material

Supplementary Material

## Data Availability

WMH-SynthSeg is publicly available and implemented in FreeSurfer: https://surfer.nmr.mgh.harvard.edu/fswiki/DownloadAndInstall https://surfer.nmr.mgh.harvard.edu/fswiki/WMH-SynthSeg Individual patient data are available to academic researchers under restricted access due to privacy and ethical restrictions, and access can be obtained by contacting the corresponding author and entering into an institutional data use agreement.
