# Supplementary material for: Detecting patterns of atrophy in cognitively impaired individuals using portable, low-field MRI
Source: Imaging Neurosci (Camb). 2026 Jan 5;4:IMAG.a.1075. doi: 10.1162/IMAG.a.1075 (PMC12770908; doi:10.1162/IMAG.a.1075)
Supplement: Supplementary Material [file IMAG.a.1075_supp.pdf]

SUPPLEMENTARY INFORMATION

Detecting Patterns of Atrophy in Cognitively Impaired Individuals Using Portable, Low-Field MRI

Ava Farnan BA[1,2], Annabel J. Sorby-Adams PhD[1,2], Jennifer Guo BS[1,2], John R. Dickson MD PhD[1], Liliana Ramirez Gomez MD[1], Amanda DeSenna FNP-BC[1], John E. Kirsch PhD[3], Joel Smith BA[4], Emma Peasley BA[4], Jeremy Ford MD MBA[5], Seyedmehdi Payabvash MD[6], Gordon Sze MD[7], Arman Fesharaki-Zadeh MD PhD[4], Christopher van Dyck MD[4], Matthew S. Rosen PhD[3], Kevin N. Sheth MD[4], J. Eugenio Iglesias PhD[3], Adam de Havenon MD MS[4], Alberto Serrano Pozo MD PhD[1], Teresa Gomez-Isla MD PhD[1], W. Taylor Kimberly MD PhD[1,2*]

1. Department of Neurology, Massachusetts General Hospital and Harvard Medical School, Boston, MA, USA
2. Center for Genomic Medicine, Massachusetts General Hospital, Boston, MA, USA
3. Athinoula A. Martinos Center for Biomedical Imaging, Massachusetts General Hospital and Harvard Medical School, Boston, MA, USA
4. Department of Neurology, Center for Brain & Mind Health, Yale New Haven Hospital and Yale School of Medicine, New Haven, CT, USA
5. Division of Neuroradiology, Department of Radiology, Massachusetts General Hospital and Harvard Medical School, Boston, MA, USA
6. Department of Radiology, Colombia University, New York, NY, USA
7. Division of Neuroradiology, Department of Radiology and Biomedical Imaging, Yale New Haven Hospital and Yale University School of Medicine, New Haven, CT, USA

* Corresponding author
W. Taylor Kimberly, MD, PhD
55 Fruit Street, Lunder 644
Boston, MA 02114 USA
wtkimberly@mgh.harvard.edu
Phone: 857-238-5644
Fax: 857-238-5601

Key Words: Low-field MRI, dementia, artificial intelligence, segmentation

Table S1. Low-field MRI (0.064 T) FLAIR sequence parameters. Parameters are reported across hardware and software versions (Hyperfine Inc) for healthy volunteer (HV), vascular comorbidities (VC), and mild cognitive impairment (MCI)/Alzheimer's disease (AD) participants.

Software Version	Hardware Version	HV (n=25)	VC (n=22)	MCI/AD (n=50)	Acquisition Time (mm:ss)	TR (ms)	TE (ms)	TI (ms)	Pixel Spacing (mm)	Slice Thickness (mm)
8.3.2	1.6	0	5	0	09:03	4000	196	1400	1.6	5.0
8.4.0	1.6	0	2	0	09:03	4000	196	1400	2.0	5.0
8.5.0	1.6	0	14	0	09:48	4000	200	1400	1.6	5.0
8.6.0	1.6	0	1	0	06:59	4000	142	1427	2.0	5.8
8.6.0	1.9	19	0	2	06:59	4000	142	1427	2.0	5.8
8.7.0	1.9	6	0	48	09:39	3500	175	1292	1.7	5.0

FLAIR – fluid attenuated inversion recovery, TE – echo time, TR – repetition time, TI – inversion time

Table S2. Left and right segmentation volumes in mm^3 from low- and high- field MRI for each structure in the vascular comorbidities cohort.

	LF Left Volume Mean (SD)	HF Left Volume Mean (SD)	LF Right Volume Mean (SD)	HF Right Volume Mean (SD)
**Global**				
WM	207785.52 (33039.92)	207785.52 (33039.92)	180347.71 (30025.60)	206613.35 (34585.35)
Cortex	228721.65 (29043.71)	226235.91 (30477.89)	223164.91 (29783.88)	227341.47 (31126.46)
WMH	N/A	N/A	N/A	N/A
**Medial Temporal**				
Hippocampus	3618.00 (560.28)	4004.89 (526.40)	3646.15 (632.65)	4137.67 (620.32)
Amygdala	1568.49 (247.04)	1748.29 (285.09)	1593.41 (335.09)	1807.31 (328.55)
**Thalamic**				
Thalamus	6315.70 (868.84)	6693.63 (1045.60)	6377.23 (1302.23)	6615.68 (1132.89)
Nucleus Accumbens	550.97 (120.52)	567.58 (127.50)	522.40 (110.26)	531.11 (116.10)
Ventral DC	3360.65 (483.16)	3898.35 (495.47)	3439.67 (597.67)	3937.33 (583.57)
**Basal Ganglia**				
Caudate	3888.09 (813.34)	3977.04 (826.86)	4026.31 (829.21)	3996.65 (796.57)
Putamen	5431.23 (718.98)	5270.62 (651.63)	5218.53 (728.88)	5303.05 (691.73)
Pallidum	1462.43 (265.97)	1433.00 (187.81)	1461.08 (342.27)	1466.06 (222.25)
**Cerebellum**				
Cerebellar WM	12263.28 (2528.35)	12635.79 (1658.70)	11566.51 (2825.13)	12672.33 (1714.77)
Cerebellar Cortex	44304.35 (8679.71)	46521.41 (5738.26)	41609.20 (11024.85)	46378.58 (5545.23)
**Ventricles**				
Lateral ventricle	20380.48 (9893.15)	17811.28 (8760.30)	19162.38 (11176.76)	17079.36 (9911.65)
Third ventricle	N/A	N/A	N/A	N/A
Fourth ventricle	N/A	N/A	N/A	N/A

DC – diencephalon, HF – high field, LF – low field, SD – standard deviation, WM – white matter, WMH – white matter hyperintensities

Table S3. Left and right segmentation volumes in mm^3 from low- and high- field MRI for each structure in the combined mild cognitive impairment and Alzheimer's disease cohort.

	LF Left Volume Mean (SD)	HF Left Volume Mean (SD)	LF Right Volume Mean (SD)	HF Right Volume Mean (SD)
**Global**				
WM	191361.07 (24241.01)	204076.22 (26498.09)	185031.81 (23489.70)	206709.31 (26564.64)
Cortex	240130.72 (29228.13)	225951.93 (23158.03)	229362.55 (27357.48)	228418.29 (22685.09)
WMH	N/A	N/A	N/A	N/A
**Medial Temporal**				
Hippocampus	3349.47 (501.92)	3366.91 (500.84)	3464.22 (471.06)	3609.76 (466.76)
Amygdala	1407.09 (268.22)	1520.36 (242.68)	1450.60 (298.85)	1621.04 (269.11)
**Thalamic**				
Thalamus	6324.64 (952.28)	6572.26 (678.73)	6408.50 (853.46)	6618.25 (721.61)
Nucleus Accumbens	506.12 (96.47)	509.04 (94.18)	471.71 (88.58)	498.54 (86.65)
Ventral DC	3933.42 (557.12)	3883.65 (668.44)	3715.15 (487.97)	3912.23 (596.82)
**Basal Ganglia**				
Caudate	4279.67 (709.36)	4093.41 (601.57)	4122.30 (636.47)	4135.00 (589.07)
Putamen	5303.47 (674.92)	4682.59 (599.81)	5084.56 (593.58)	4752.89 (579.05)
Pallidum	1518.00 (212.29)	1534.59 (215.82)	1508.43 (226.46)	1566.79 (225.40)
**Cerebellum**				
Cerebellar WM	12263.28 (2528.35)	13181.50 (1812.83)	12524.06 (2299.39)	13158.22 (1878.46)
Cerebellar Cortex	47397.86 (8564.59)	49667.28 (4932.85)	45524.69 (7620.47)	49581.45 (4933.25)
**Ventricles**				
Lateral ventricle	36552.07 (14775.45)	34093.41 (15147.21)	30510.89 (12082.86)	28702.05 (12414.11)
Third ventricle	N/A	N/A	N/A	N/A
Fourth ventricle	N/A	N/A	N/A	N/A

DC – diencephalon, HF – high field, LF – low field, SD – standard deviation, WM – white matter, WMH – white matter hyperintensities

Table S4. Validation of low- to high- field MRI segmentation volumes in all cohorts.

	Pearson r<br>Mean (95% CI)	ASPD<br>Median [IQR]	Dice<br>Median [IQR]
**Global**			
WM	0.91 (0.87, 0.94)	1.31 [0.67, 2.08]	0.79 [0.77, 0.81]
Cortex	0.92 (0.88, 0.95)	3.15 [1.44, 6.45]	0.73 [0.70, 0.75]
WMH	0.88 (0.83, 0.92)	7.56 [4.15, 12.00]	0.52 [0.45, 0.62]
**Medial Temporal**			
Hippocampus	0.87 (0.81, 0.91)	6.91 [2.09, 10.54]	0.80 [0.77, 0.82]
Amygdala	0.89 (0.84, 0.93)	9.42 [3.90, 15.88]	0.82 [0.80, 0.85]
**Thalamic**			
Thalamus	0.82 (0.74, 0.87)	6.04 [2.05, 11.47]	0.84 [0.81, 0.87]
Nucleus Accumbens	0.82 (0.74, 0.87)	9.90 [5.91, 15.12]	0.76 [0.72, 0.79]
Ventral DC	0.59 (0.44, 0.71)	6.46 [3.29, 9.73]	0.78 [0.75, 0.81]
**Basal Ganglia**			
Caudate	0.82 (0.74, 0.88)	4.76 [1.54, 8.56]	0.82 [0.78, 0.84]
Putamen	0.75 (0.65, 0.83)	10.07 [3.69, 18.25]	0.84 [0.81, 0.86]
Pallidum	0.62 (0.48, 0.73)	8.46 [4.28, 11.89]	0.75 [0.70, 0.78]
**Cerebellum**			
Cerebellar WM	0.73 (0.60, 0.82)	6.00 [3.58, 9.77]	0.79 [0.77, 0.80]
Cerebellar Cortex	0.86 (0.79, 0.91)	4.52 [1.55, 7.46]	0.84 [0.83, 0.85]
**Ventricles**			
Lateral ventricle	0.99 (0.99, 0.99)	6.43 [3.12, 13.44]	0.86 [0.81, 0.90]
Third ventricle	0.96 (0.94, 0.97)	2.50 [1.28, 4.10]	0.78 [0.71, 0.83]
Fourth ventricle	0.88 (0.83, 0.92)	5.24 [3.05, 8.83]	0.76 [0.69, 0.79]

ASPD – absolute symmetrized percent difference, CI – confidence interval, DC – diencephalon, IQR – interquartile range, WM – white matter, WMH – white matter hyperintensities

Table S5. Validation of low- to high- field MRI segmentation volumes in the healthy volunteer cohort.

	Pearson r<br>Mean (95% CI)	ASPD<br>Median [IQR]	Dice<br>Median [IQR]
**Global**			
WM	0.97 (0.93, 0.99)	5.86 [4.11, 8.72]	0.82 [0.81, 0.82]
Cortex	0.95 (0.89, 0.98)	1.97 [1.61, 2.93]	0.76 [0.75, 0.77]
WMH	0.72 (0.45, 0.87)	5.55 [1.44, 8.92]	0.47 [0.41, 0.53]
**Medial Temporal**			
Hippocampus	0.89 (0.76, 0.95)	3.42 [1.86, 5.48]	0.80 [0.78, 0.82]
Amygdala	0.72 (0.45, 0.87)	3.68 [1.63, 7.49]	0.82 [0.81, 0.84]
**Thalamic**			
Thalamus	0.80 (0.59, 0.91)	8.92 [3.62, 12.25]	0.82 [0.78, 0.85]
Nucleus Accumbens	0.85 (0.68, 0.93)	6.29 [2.81, 9.57]	0.79 [0.76, 0.82]
Ventral DC	0.80 (0.59, 0.91)	13.07 [6.34, 21.64]	0.78 [0.74, 0.80]
**Basal Ganglia**			
Caudate	0.87 (0.72, 0.94)	6.32 [4.07, 8.84]	0.84 [0.82, 0.85]
Putamen	0.94 (0.87, 0.97)	1.70 [0.98, 3.72]	0.87 [0.86, 0.89]
Pallidum	0.66 (0.36, 0.84)	9.32 [3.27, 15.22]	0.76 [0.72, 0.78]
**Cerebellum**			
Cerebellar WM	0.74 (0.47, 0.88)	6.53 [1.67, 10.84]	0.80 [0.79, 0.81]
Cerebellar Cortex	0.87 (0.71, 0.94)	6.49 [2.14, 7.99]	0.85 [0.85, 0.86]
**Ventricles**			
Lateral ventricle	0.98 (0.95, 0.99)	10.00 [7.41, 16.28]	0.79 [0.76, 0.82]
Third ventricle	0.70 (0.42, 0.86)	5.13 [2.16, 6.46]	0.71 [0.59, 0.77]
Fourth ventricle	0.95 (0.89, 0.98)	3.92 [2.80, 5.53]	0.79 [0.76, 0.82]

ASPD – absolute symmetrized percent difference, CI – confidence interval, DC – diencephalon, IQR – interquartile range, WM – white matter, WMH – white matter hyperintensities

Table S6. Validation of low- to high- field MRI segmentation volumes in the vascular comorbidities cohort.

	Pearson r<br>Mean (95% CI)	ASPD<br>Median [IQR]	Dice<br>Median [IQR]
**Global**			
WM	0.93 (0.84, 0.97)	12.43 [8.96, 13.80]	0.79 [0.75, 0.81]
Cortex	0.93 (0.84, 0.97)	3.56 [1.85, 5.26]	0.74 [0.70, 0.76]
WMH	0.92 (0.81, 0.97)	3.22 [0.73, 7.33]	0.53 [0.45, 0.64]
**Medial Temporal**			
Hippocampus	0.90 (0.77, 0.96)	11.69 [7.52, 15.32]	0.79 [0.77, 0.81]
Amygdala	0.92 (0.81, 0.97)	9.95 [6.15, 16.99]	0.82 [0.79, 0.85]
**Thalamic**			
Thalamus	0.79 (0.55, 0.91)	7.16 [4.73, 13.48]	0.82 [0.77, 0.85]
Nucleus Accumbens	0.86 (0.69, 0.94)	8.44 [5.67, 11.88]	0.76 [0.68, 0.82]
Ventral DC	0.73 (0.45, 0.88)	12.29 [8.91, 21.38]	0.77 [0.72, 0.81]
**Basal Ganglia**			
Caudate	0.94 (0.86, 0.98)	3.34 [2.36, 6.63]	0.79 [0.73, 0.83]
Putamen	0.89 (0.75, 0.95)	4.37 [1.90, 5.85]	0.82 [0.81, 0.85]
Pallidum	0.79 (0.55, 0.91)	7.60 [3.93, 15.55]	0.72 [0.68, 0.77]
**Cerebellum**			
Cerebellar WM	0.64 (0.21, 0.86)	5.78 [3.38, 9.98]	0.78 [0.75, 0.79]
Cerebellar Cortex	0.88 (0.68, 0.96)	3.46 [1.38, 5.46]	0.83 [0.81, 0.83]
**Ventricles**			
Lateral ventricle	0.97 (0.93, 0.99)	10.76 [8.26, 15.21]	0.84 [0.81, 0.86]
Third ventricle	0.82 (0.61, 0.92)	3.94 [2.06, 8.86]	0.77 [0.67, 0.81]
Fourth ventricle	0.93 (0.84, 0.97)	8.79 [5.73, 11.43]	0.66 [0.63, 0.74]

ASPD – absolute symmetrized percent difference, CI – confidence interval, DC – diencephalon, IQR – interquartile range, WM – white matter, WMH – white matter hyperintensities

Table S7. Validation of low- to high- field MRI segmentation volumes in the combined mild cognitive impairment and Alzheimer's disease cohort.

	Pearson r<br>Mean (95% CI)	ASPD<br>Median [IQR]	Dice<br>Median [IQR]
**Global**			
WM	0.90 (0.83, 0.94)	1.31 [0.67, 2.08]	0.78 [0.76, 0.79]
Cortex	0.93 (0.88, 0.96)	3.15 [1.44, 6.45]	0.70 [0.69, 0.72]
WMH	0.83 (0.72, 0.90)	7.56 [4.15, 12.00]	0.54 [0.47, 0.63]
**Medial Temporal**			
Hippocampus	0.79 (0.66, 0.88)	6.91 [2.09, 10.54]	0.80 [0.77, 0.82]
Amygdala	0.90 (0.83, 0.94)	9.42 [3.90, 15.88]	0.83 [0.81, 0.86]
**Thalamic**			
Thalamus	0.76 (0.61, 0.86)	6.04 [2.05, 11.47]	0.86 [0.83, 0.88]
Nucleus Accumbens	0.68 (0.50, 0.81)	9.90 [5.91, 15.12]	0.75 [0.72, 0.79]
Ventral DC	0.59 (0.37, 0.75)	6.46 [3.29, 9.73]	0.79 [0.76, 0.82]
**Basal Ganglia**			
Caudate	0.76 (0.61, 0.86)	4.76 [1.54, 8.56]	0.81 [0.78, 0.83]
Putamen	0.48 (0.23, 0.67)	10.07 [3.69, 18.25]	0.82 [0.80, 0.84]
Pallidum	0.59 (0.37, 0.75)	8.46 [4.28, 11.89]	0.77 [0.71, 0.80]
**Cerebellum**			
Cerebellar WM	0.76 (0.57, 0.87)	6.00 [3.58, 9.77]	0.78 [0.77, 0.79]
Cerebellar Cortex	0.84 (0.70, 0.92)	4.52 [1.55, 7.46]	0.83 [0.82, 0.85]
**Ventricles**			
Lateral ventricle	0.99 (0.98, 0.99)	6.43 [3.12, 13.44]	0.90 [0.88, 0.92]
Third ventricle	0.96 (0.93, 0.98)	2.50 [1.28, 4.10]	0.82 [0.78, 0.85]
Fourth ventricle	0.86 (0.76, 0.92)	5.24 [3.05, 8.83]	0.76 [0.70, 0.78]

ASPD – absolute symmetrized percent difference, CI – confidence interval, DC – diencephalon, IQR – interquartile range, WM – white matter, WMH – white matter hyperintensities

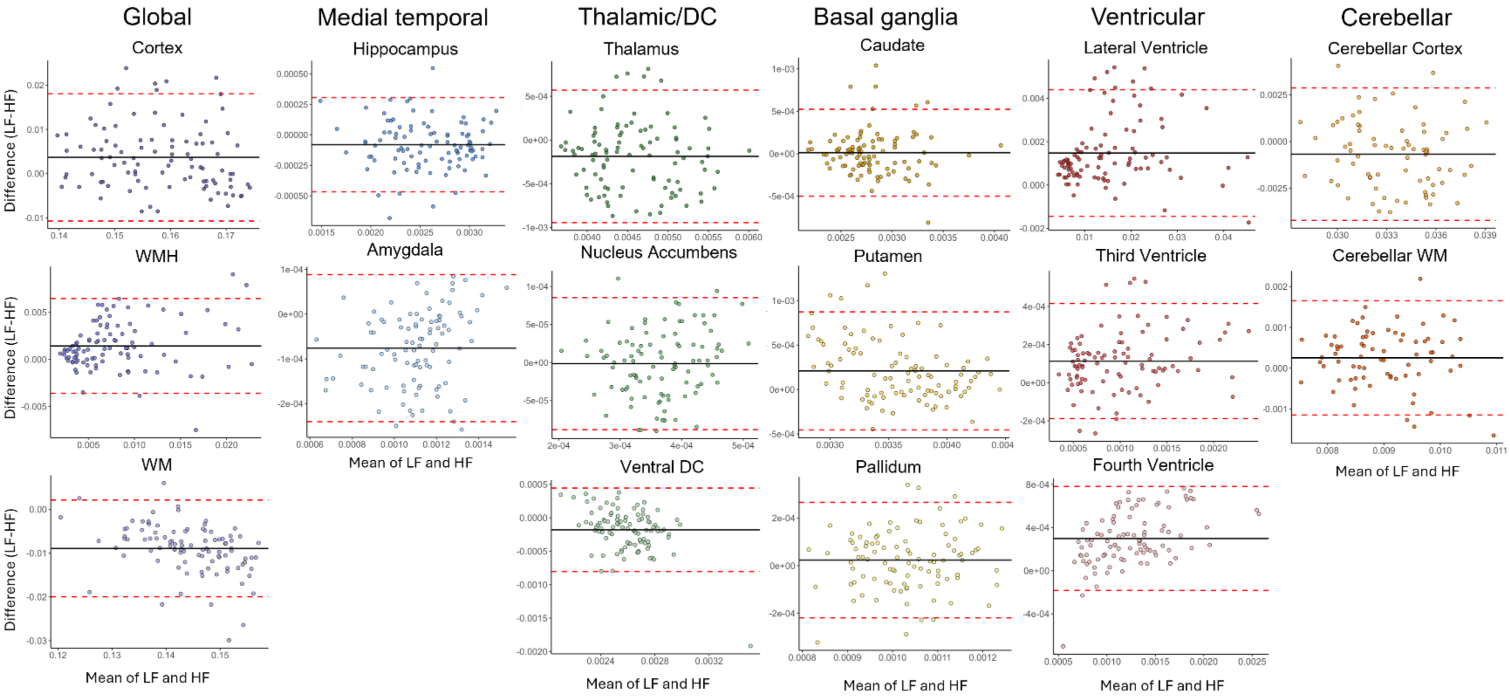

Figure S1. Bland-Altman plots for all segmentation volumes across all cohorts. Bland-Altman plots comparing low- and high- field segmented brain volumes. Each brain region is grouped by tissue class into global, medial temporal, thalamic/DC, basal ganglia, ventricular, and cerebellar. The mean LF and HF volumes are plotted against their difference (LF - HF). The solid black line indicates the mean difference, and the red dashed lines denote the 95% limits of agreement. Abbreviations are defined as DC – diencephalon, WM – white matter, WMH – white matter hyperintensities.

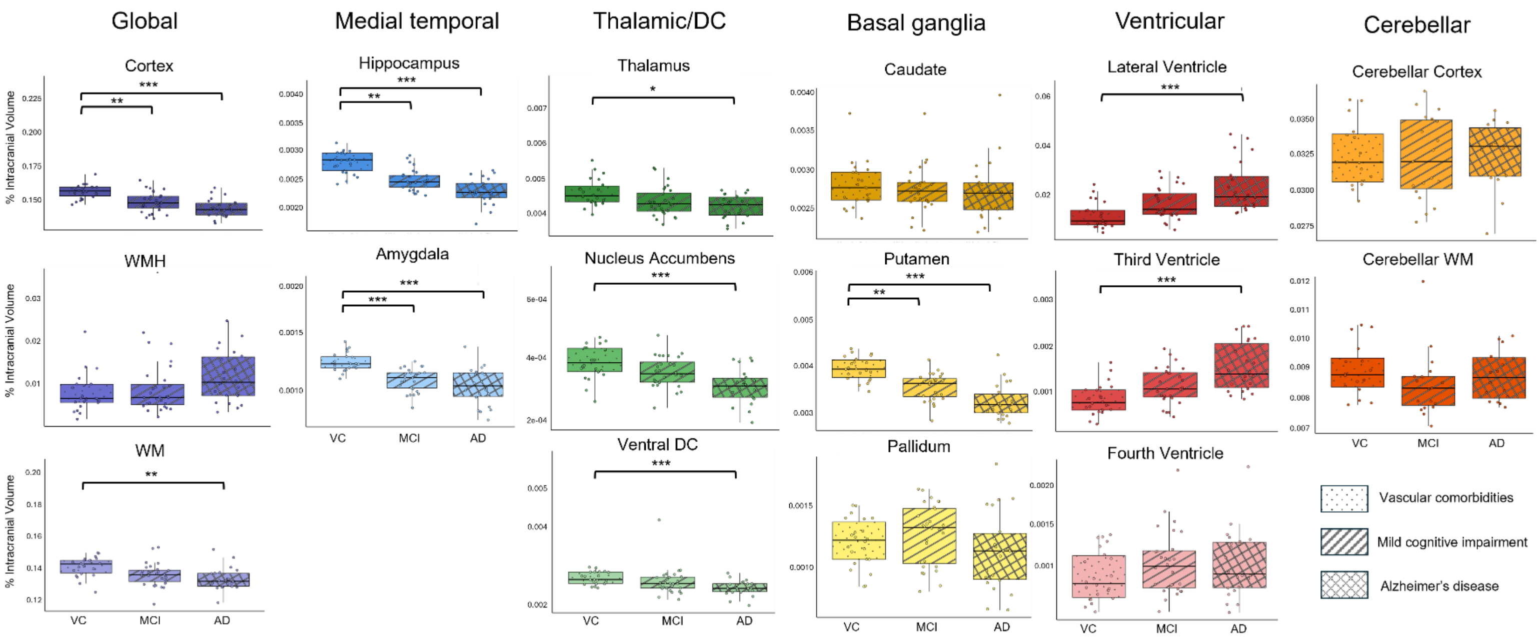

Figure S2. Comparison of high-field segmented brain volumes across all cohorts. Box plots comparing segmented brain volumes between the vascular comorbidities (VC), mild cognitive impairment (MCI), and Alzheimer's disease (AD) cohorts. Each brain region is grouped by tissue class into global, medial temporal, thalamic/DC, basal ganglia, ventricular, and cerebellar. Individual data points are overlaid on each box plot to show the distribution of individual measurements. All volumes are adjusted by intracranial volume and reported as percent of intracranial volume. Significance is denoted as * $p < 0.05$, ** $p < 0.01$, and *** $p < 0.001$. Abbreviations are defined as DC – diencephalon, WM – white matter, WMH – white matter hyperintensities

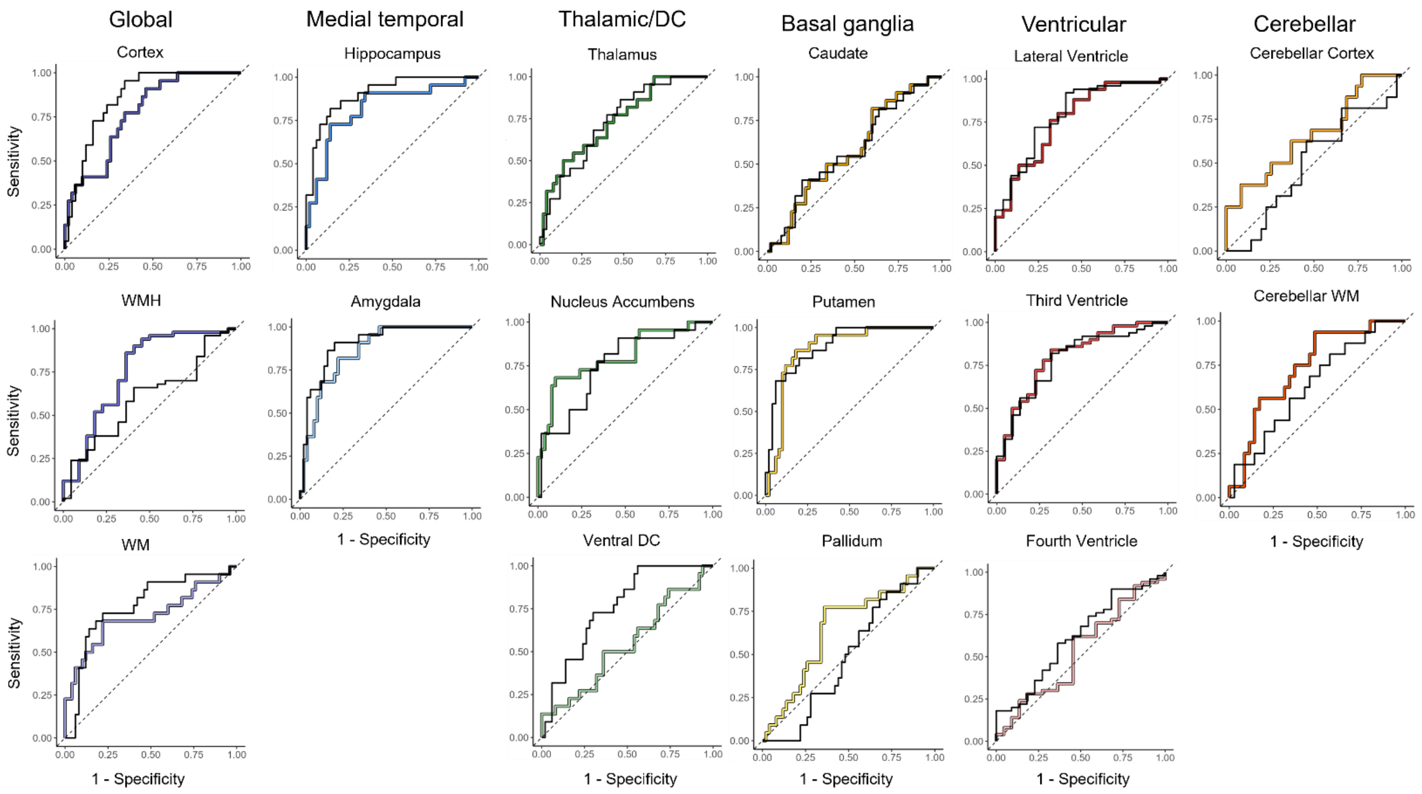

Figure S3. ROC curves for classification performance of low- and high- field MRI segmentation volumes. Receiver operating characteristic (ROC) curves illustrating the ability of low-field MRI derived brain volumes to classify participants between the vascular comorbidities (VC) cohort and the mild cognitive impairment/Alzheimer's disease (MCI/AD) cohort. Each brain region is grouped by tissue class into global, medial temporal, thalamic/DC, basal ganglia, ventricular, and cerebellar. For a given brain volume, the colored ROC curve represents low-field values, while the black ROC curve represents high-field values. The dashed diagonal line represents chance-level classification (AUC = 0.5). Abbreviations are defined as DC – diencephalon, WM – white matter, WMH – white matter hyperintensities.

Table S8. Comparison of low- and high-field MRI AUC values for all segmentation volumes

	LF AUC (95% CI)	HF AUC (95% CI)	P Value
**Global**			
WM	0.71 (0.56, 0.86)	0.77 (0.64, 0.89)	0.307
Cortex	0.77 (0.66, 0.88)	0.86 (0.77, 0.94)	0.088
WMH	0.76 (0.62, 0.89)	0.60 (0.46, 0.74)	0.001
**Medial Temporal**			
Hippocampus	0.82 (0.70, 0.93)	0.90 (0.83, 0.98)	0.014
Amygdala	0.86 (0.78, 0.94)	0.90 (0.83, 0.97)	0.274
**Thalamic**			
Thalamus	0.74 (0.61, 0.86)	0.73 (0.61, 0.85)	0.944
Nucleus Accumbens	0.80 (0.68, 0.92)	0.75 (0.63, 0.87)	0.337
Ventral DC	0.46 (0.31, 0.61)	0.76 (0.65, 0.87)	< 0.001
**Basal Ganglia**			
Caudate	0.58 (0.44, 0.72)	0.58 (0.44, 0.73)	0.987
Putamen	0.87 (0.78, 0.96)	0.89 (0.81, 0.97)	0.659
Pallidum	0.65 (0.51, 0.78)	0.49 (0.35, 0.62)	0.033
**Cerebellum**			
Cerebellar WM	0.74 (0.59, 0.88)	0.64 (0.48, 0.80)	0.229
Cerebellar Cortex	0.66 (0.49, 0.83)	0.50 (0.33, 0.67)	0.012
**Ventricles**			
Lateral ventricle	0.77 (0.64, 0.89)	0.79 (0.68, 0.91)	0.107
Third ventricle	0.80 (0.69, 0.91)	0.78 (0.67, 0.90)	0.581
Fourth ventricle	0.46 (0.31, 0.61)	0.61 (0.47, 0.76)	< 0.001

AUC – Area Under the Curve, CI – confidence interval, DC – diencephalon, HF – high field, LF – low field, WM – white matter, WMH – white matter hyperintensities
